# Trends in Antihypertensive Medication Use and Blood Pressure Control in Adults Aged 66–79: Results of the National Examination Surveys DEGS1 and Study on Health of Older People Gesundheit 65+

**DOI:** 10.1111/jch.70250

**Published:** 2026-04-11

**Authors:** Giselle Sarganas, Julia Büschges, Dinara Yessimova, Markus van der Giet, Judith Fuchs, Hannelore Neuhauser

**Affiliations:** ^1^ Department of Epidemiology and Health Monitoring Robert Koch Institute Berlin Germany; ^2^ DZHK (German Centre for Cardiovascular Research), partner site Berlin Berlin Germany; ^3^ Medizinische Klinik für Nephrologie und internistische Intensivtherapie Charité – Universitätsmedizin Berlin Berlin Germany

## Abstract

Antihypertensive medication use is highly prevalent among older adults, with treatment patterns evolving over time. This study examines trends in antihypertensive medication use in the general population and among treated hypertensive individuals aged 66–79 years, based on two nationwide German population‐based surveys conducted in 2008–2011 and 2021–2023. Data were derived from the German Health Interview and Examination Survey for Adults (DEGS1; *n* = 1733) and the Study on Health of Older People in Germany (Gesundheit 65+; *n* = 680). Both surveys provide comparable information on antihypertensive drug classes—angiotensin‐converting enzyme (ACE) inhibitors, angiotensin receptor blockers (ARBs), calcium channel blockers (CCBs), diuretics, beta‐blockers, and others—as well as blood pressure (BP) control (<140/90 mmHg) assessed using standardized oscillometric measurements. The prevalence of antihypertensive medication use in the general population remained relatively stable between 2008–2011 (68.7%) and 2021–2023 (64.5%). However, treatment patterns changed, with decreased use of diuretics and ACE inhibitors and increased use of ARB. Among treated hypertensive individuals, BP control remained largely unchanged at approximately two‐thirds (69.1% vs. 63.6%). Notably, there was a shift from single‐pill combination therapy toward monotherapy, accompanied by a decline in BP control among women (73.8%–58.0%). Overall, approximately two‐thirds of adults aged 66–79 years in Germany use antihypertensive medication. Over the past decade, BP control has not improved among treated hypertensive men and has worsened among women. Furthermore, the use of single‐pill combination therapies remains low and has declined among treated hypertensive individuals.

## 1 Introduction

Hypertension is a leading risk factor for cardiovascular (CV) morbidity and mortality worldwide [1, 2]. A continuous, log‐linear relationship links blood pressure (BP) to CV events, with risk beginning to rise at systolic BP (SBP) levels at least as low as 115 mmHg [3]. To reflect this in the European Society of Cardiology (ESC) Guidelines, an additional category termed “elevated BP” has been formally introduced in the 2024 Guidelines, defined by a SBP between 120 and 139 mmHg or diastolic BP (DBP) between 70 and 89 mmHg [4, 5]. At these BP levels, antihypertensive therapy may be considered after 3 months of lifestyle modification without reducing BP, or if a person is at high risk of CV events [4]. The ESC 2024 Guidelines recommends further five major classes of antihypertensive medications with strong evidence for reducing CV events through lowering BP: angiotensin‐converting enzyme (ACE) inhibitors, angiotensin receptor blockers (ARBs), calcium channel blockers (CCBs), diuretics (thiazides and thiazide‐like diuretics), and beta‐blockers. Further, an initial treatment with a single‐pill combination of two major drug classes is recommended [4]. This recommendation reflects the assumption that combining medications from different drug classes yields synergistic effects rather than increasing the dose of a single medication for a greater BP reduction [4, 5].

In 1998, Germany showed higher BP levels and lower treatment and control rates compared to other Western countries [6]. One decade later, between 2008 and 2011, there was a marked improvement: the average level of population BP in Germany had significantly decreased, and the proportion of controlled hypertension more than doubled with a notable improvement in the use of antihypertension medication [6]. Accompanying this trend was a shift from monotherapy toward polytherapy. The high rate of polytherapy in Germany (67%) in 2008–2011 exceeded that the US (48%), England (54%), and France (56%) [6].

As new scientific evidence and updated guidelines continue to shape trends in hypertension management, it is essential to keep monitoring patterns in antihypertensive medication use and BP control. This study aims to investigate recent trends in the use of antihypertensives and BP control among adults 66–79 years old in Germany from 2008–2011 to 2021–2023 and to highlight potential areas for improvement in clinical practice.

## 2 Materials and Methods

Two nationwide population‐based studies, the German National Health Interview and Examination Survey for Adults (Studie zur Gesundheit Erwachsener in Deutschland, abbreviated as DEGS1) and the Study on Health of Older People in Germany (abbreviated as Gesundheit 65+), provide comparable data on BP measured according to a standardized protocol, on the use of antihypertensive medication in the past 7 days, hypertension treatment and control among older individuals in Germany. In both studies, population registries were used to set up a two‐stage sampling process [7, 8]. In the DEGS1 study, adults aged 18–79 years underwent medical interviews and examinations in examination centers across Germany from 2008 to 2011. This analysis included 1733 older adults aged 66–79 years. DEGS1 was approved by the ethics committee of the Charité University Hospital, Berlin (No. EA2/047/08). The study Gesundheit 65+, carried out between 2021 and 2023, included people aged 65 years and older in Germany. This analysis included 680 non‐institutionalized participants aged 66–79 years who were interviewed and examined at home. The study was approved by the ethics committee of the medical association in Berlin (Berliner Ärztekammer, Eth‐50/19). Participants provided written informed consent before the interviews and examinations in both studies. Detailed information on sampling design, study procedures, and quality control, among other aspects, for both studies has been published previously [7, 8].

### 2.1 Measurements

In both studies, information on current medication intake (7 days previous interview) was obtained in computer‐assisted personal interviews [9]. Barcode‐scanned medication packages or when missing medication names were used to derive Anatomical Therapeutic Chemical (ATC) codes. Six classes of medication with anti‐hypertensive main effect were differentiated by using ATC codes: diuretics, beta‐blockers, CCBs, ACE inhibitors, ARBs, and other antihypertensive drugs (Table S1). Monotherapy refers to treatment with antihypertensives from a single active ingredient. Combination therapy or polytherapy was defined as the use of antihypertensive medication with several antihypertensive agents. This includes the use of single‐pill combinations containing several antihypertensive agents. When investigating the use of antihypertensive medication by class, combination therapy users were counted in each antihypertensive class corresponding to the antihypertensive agents included in their therapy.

In both studies, brachial BP was measured using automated oscillometric devices (DEGS1: Datascope Accutorr Plus, Mahwah, New Jersey, USA; Gesundheit 65+: Mobil‐O‐Graph, IEM GmbH, Aachen, Germany) [7, 8]. Three BP measurements with appropriately sized upper arm cuffs were taken in a sitting position after 5 min of rest. Individual SBP and DBP were calculated as the arithmetic mean of the second and third measurements. Detailed information on BP measurement in both studies has been published [7, 8]. A calibration formula [10] was employed to compensate for differences between the two oscillometric devices.

Hypertension was defined based on either hypertensive BP values (SBP ≥140 mmHg and/or DBP ≥90 mmHg) or treatment. Treatment was defined as the use of medication with an antihypertensive main effect from any of the classes listed above by participants who reported a physician diagnosis of hypertension at baseline or follow‐up. Among the treated individuals, hypertension was considered controlled if measured SBP was <140 mmHg and DBP <90 mmHg.

### 2.2 Covariates

Weight in kilos was measured with a calibrated scale (DEGS1: Column Scale 930, seca; Gesundheit 65+: Seca 208, seca, Hamburg/Germany) and a portable stadiometer was used to obtain height in cm (DEGS1: Holtain Ltd., UK; Gesundheit 65+: Seca 213, seca, Hamburg/Germany). Participants with a BMI of at least 30 kg/m^2^ were considered having obesity. Participants were classified as having diabetes if they reported a diagnosis of diabetes or reported current use of antidiabetic medication (ATC code: A10). Participants reported their current smoking behavior and any coronary heart disease diagnosis during the past 12 months via questionnaire. The Comparative Analysis of Social Mobility in Industrial Nations (CASMIN) classification was used to describe the participants’ level of education [11].

### 2.3 Statistical Analysis

Frequencies of categorical variables are denoted as prevalence when the denominator is the population and as proportion if they refer to selected groups, for example, to treated hypertensives. Both are reported with 95% confidence intervals (CIs). For continuous variables such as BP in mmHg, we report means and 95% CI.

To account for deviations of both samples from the general population and changes in the general population between 2008 and 2021, statistical weights (date of reference: December 31 2020), including age, sex, nationality, and educational level, were applied [8]. Analyses were conducted using STATA, Version 17 (Stata Corp. 2021. Stata Statistical Software: Release 17. College Station, TX: StataCorp LLC.). 95% CIs were estimated to inform on the precision of our estimates. The Wald test was used to test for statistical significance of differences between the two studies with significance set at *α* = 0.05 (two‐sided). Analyses and reporting followed the Strengthening the Reporting of Observational Studies in Epidemiology guidelines [12].

## 3 Results

**FIGURE 1:**
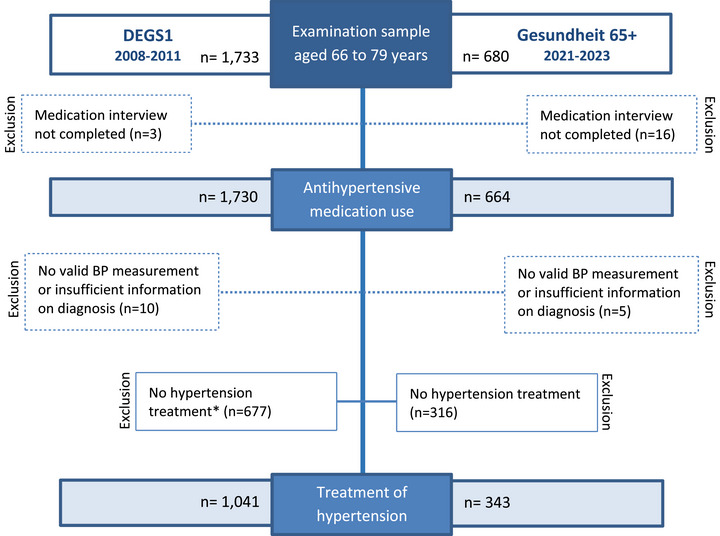
Flow chart of sample selection for analysis of antihypertensive medication use in the DEGS1 and Gesundheit65+ studies. *No hypertension treatment: Participants without hypertension, unaware of hypertension or with untreated hypertension. BP, blood pressure.

Figure 1 provides an overview of the data selection process. From the 1733 DEGS1 and 680 Gesundheit 65+ participants, we excluded those without completed medication interview (3 DEGS1 and 16 Gesundheit 65+). Our analysis consists of two parts: First, we analyzed the use of antihypertensive medication in the general population in 1730 older adults participating in the DEGS1 (2008–2011) and 664 participants in Gesundheit 65+ study (2021–2023). In the second part, we focus on treated hypertension, that is, on 1041 DEGS1 participants and 343 Gesundheit 65+ participants, and analyze their use of antihypertensive medication and BP control.

### 3.1 Antihypertensive Medication Use in the General Population

Table 1 presents key indicators in DEGS1 and Gesundheit 65+. Both samples included slightly more women than men (DEGS1: 54.6%, Gesundheit 65+: 53.8%). Participants of Gesundheit 65+ (17.0%) had obtained higher levels of education compared to the DEGS1 (10.9%) study participants 15 years ago. A higher proportion of participants in the Gesundheit 65+ study (14.1%) smoked compared with participants in DEGS1 (9.2%). Other explored risk factors and comorbidities were similar in both samples. In 2023, the proportion of participants with a hypertensive BP measurement was higher compared to participants from 2010 (DEGS1: 28.4%, Gesundheit 65+: 33.5%), while elevated BP was more prevalent in 2010. In both studies, around two thirds of participants used medication with antihypertensive main effects (DEGS1: 68.7%, Gesundheit 65+: 64.5%). Among users of antihypertensive medication, the vast majority had hypertension: 88.1% (85.3–90.8) in DEGS1 and 80.7% in Gesundheit 65+ (75.4–85.3).

**TABLE 1:** Key characteristics of older adults aged 66–79 years in the DEGS (2008–2011) and Gesundheit 65+ (2021–2023) studies in Germany.

	% Of study participants (95% CI)		
	DEGS (2008–2011)	Gesundheit 65+ (2021–2023)	
	*n*=1730	*n*=664	*p* difference
Sex			n.s.
Men	45.37 (42.80–47.96)	46.16 (42.05–50.33)	
Women	54.63 (52.04–57.20)	53.84 (49.67–57.95)	
Age (years)			n.s.
66–69	35.15 (32.57–37.83)	34.00 (29.36–38.96)	
70–79	64.85 (62.17–67.43)	66.00 (61.04–70.64)	
Education^c^			0.00
Low	66.26 (62.43–69.88)	46.55 (41.02; 52.18)	
Medium	22.88 (20.10–25.92)	36.41 (31.21–41.94)	
High	10.86 (9.06–12.96)	17.04 (14.20–20.31)	
Risk factors			
Current smoking^a^	9.24 (7.64–11.13)	14.07 (10.53–18.55)	0.02
Obesity	35.81 (32.69–39.06)	30.22 (25.81–35.03)	n.s.
Comorbidities			
Diabetes^b^	19.65 (17.11–22.46)	17.90 (14.19–22.34)	n.s.
CHD in past 12 months	6.30 (4.91–8.04)	8.08 (5.72–11.31)	n.s.
BP measurement			0.01
Non‐elevated: <120/70 mmHg	15.97 (13.73–18.50)	19.37 (16.13–23.08)	
Elevated: 120–139/70–89 mmHg	55.63 (52.72–58.50)	46.70 (41.71–51.76)	
Hypertensive: ≥140/90 mmHg	28.40 (25.45–31.56)	33.92 (29.73–38.80)	
Antihypertensive drug use	68.73 (65.82–71.50)	64.51 (59.37–69.33)	n.s.

Abbreviations: BP, blood pressure; CI, confidence interval; CHD, coronary heart disease.

^a^ Daily or occasional smoking.

^b^ Diagnosis or intake of antidiabetics.

^c^ Using the Comparative Analysis of Social Mobility in Industrial Nations classification.

**FIGURE 2:**
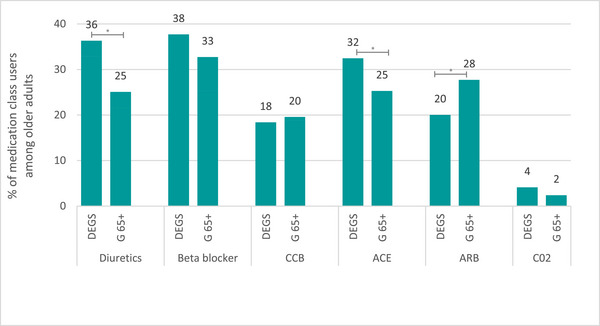
Prevalence of antihypertensive medication use among older adults aged 66–79 years in Germany in the DEGS (2008–2011, *n*=1730) and Gesundheit65+ (G65+) studies (2021–2023, *n*=664) *p < 0.05. ACE, angiotensin‐converting enzyme; ARB, angiotensin receptor blockers; CCB, calcium channel blockers; C02, Antihypertensive with Anatomical Therapeutic Chemical Code C02 (i.e., antiadrenergic agents).

The use of antihypertensive medication by class is depicted in Figure 2. From 2008 to 2023, beta‐blockers remained the most commonly used antihypertensive agents (DEGS1: 38%, Gesundheit 65+: 33%). Meanwhile, the use of diuretics statistically significantly decreased from 36.3% to 25.1%. Further analysis revealed this reduction to be observed equally in both men and women (Table S2). The use of ACE inhibitors decreased statistically significant as well; however, this reduction was mostly driven by men from 37.0% (95% CI 33.1–41.2) to 27.4% (95% CI 21.9–33.7) (*p* < 0.05). Another significant change in the use of antihypertensive medication was in the group of ARBs, where a statistically significantly increase from 20.0% to 27.7% was observed. This rise was primarily driven by men again, from 18.2% (95% CI 15.3–21.6) to 30.8% (25.7–36.5) (*p* < 0.05). The use of CCB and antihypertensives with ATC code C02 (i.e., antiadrenergic agents) remained unchanged at around 19% and 2%, respectively. In both studies, most ARB, CCB, and antihypertensives with ATC code C02 users were receiving treatment for hypertension; while a smaller share of users of diuretics, beta‐blockers and ACE inhibitors users were on hypertension treatment.

### 3.2 Antihypertensive Medication and BP Control in Treated Hypertensives

Table 2 displays, among treated hypertensives, mean SBP and DBP and proportions of BP control by monotherapy and polytherapy treatment (see Table S3 for results stratified by sex). Changes in proportions with antihypertensive monotherapy and polytherapy use have been observed; however, they were not statistically significant: monotherapy increased from 25.7% in 2010 to 32.6% in 2023, while polytherapy use correspondingly decreased from 74.3% to 67.4%. Examining the use of polytherapy with its two components in detail, that is, single pill combination and several pills, we observed that the monotherapy‐polytherapy shift was due to a statistically significant decrease in single pill combination use (from 14.5% to 6.6%), mirroring the increase in monotherapy (from 25.7% to 32.6%). In contrast, the use of several pills for treating hypertension remained unchanged at 60%. Overall mean BP levels among treated hypertensives were stable from 2010 to 2023. However, mean SBP among men decreased slightly but statistically significant from 134.9 (95% CI 133.3–136.7) to 128.7 (126.0‐131.5) mmHg in the observed period (Table S3). This decrease was observed among both mono‐ and polytherapy male users. The overall BP control in the observed period slightly decreased from 69.1% to 63.6%, being to some extent higher among users of polytherapy (n.s.). Among women, BP control declined statistically significantly by 15.8% points, from 73.8% (68.8–78.2) in 2010 to 58.0% (47.4–67.9) in 2023. Among men, slightly increased from 63.0% (57.0–68.5) to 69.8% (61.1–77.2); however, not statistically significantly (Table S3).

**TABLE 2:** Mean BP and proportion of BP control among participants aged 66–79 years with treated hypertension in Germany in the DEGS1 (2008–2011, *n*=1041) and G65+ (Gesundheit 65+ 2021–2023, *n*=343) studies, with 95% confidence intervals (CI).

				Polytherapy			
		Monotherapy		Single pill combination		Several pills	
	Total		95% CI		95% CI		95% CI
DEGS1 (2010)	100%	25.70	22.57–29.10	**14.47**	12.11–17.20	59.83	56.00–63.53
G65+ (2023)	100%	32.56	26.21–39.62	**6.64**	4.02–10.76	60.8	53.97–67.23
Mean SBP (in mmHg)							
DEGS1 (2010)	132.63	134.85	132.82–136.89	133.80	131.15–136.46	131.40	129.80–133.01
G65+ (2023)	131.28	133.24	128.44–138.03	129.65	124.90–134.39	130.39	127.52–133.25
Mean DBP (in mmHg)							
DEGS1 (2010)	81.78	83.45	82.08–84.82	83.30	81.37–85.23	80.71	79.67–81.74
G65+ (2023)	81.85	84.23	81.84–86.62	79.29	74.42–84.17	80.82	79.20–82.44
BP control (in %)^a^							
DEGS1 (2010)	69.07	62.71	54.29–70.43	64.62	53.78–74.15	72.88	68.05–77.22
G65+ (2023)	63.63	60.56	48.23–71.68	67.23	37.75–87.41	64.88	56.59–72.36

*Note*: Weighted to the population 2020. Values printed bold if differences between studies statistically significant (*p*<0.05).

^a^ BP control: <140/90 mmHg

**FIGURE 3:**
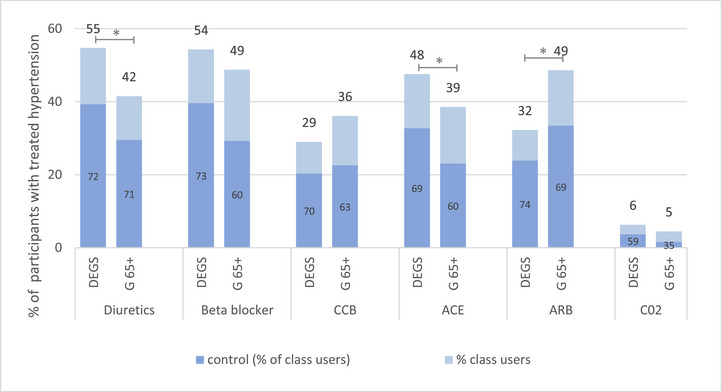
Antihypertensive treatment and control among older adults aged 66–79 years with treated hypertension in Germany in the DEGS (2008–2011, *n*=1041) and Gesundheit65+ (G65+) (2021–2023, *n*=343) studies. *p < 0.05. ACE, angiotensin‐converting enzyme; ARB, angiotensin receptor blockers; CCB, calcium channel blockers; C02, Antihypertensive with Anatomical Therapeutic Chemical Code C02.

Figure 3 shows antihypertensive treatment and control in treated hypertensives stratified by antihypertensive agent. In the DEGS1 study (2008–2011), diuretics (55%) and beta‐blockers (54%) were the most commonly used antihypertensive agents among treated hypertensives. Twelve years later (Gesundheit 65+, 2021–2023), diuretics use has decreased to 41% (*p* < 0.05), making ARB (49%) and beta‐blockers (49%) the other most commonly used classes of antihypertensive medication. Overall control rates per drug class used remained largely unchanged across the two study periods.

## 4 Discussion

This study investigated the trends in the use of antihypertensive medication between 2010 and 2023 in Germany, and assessed BP control rates among treated men and women with hypertension. Although diuretics were the most used class of antihypertensive medication in 1998 (43%) [6], beta‐blockers became most prevalent in 2010 (38%), remaining the leading group in 2023 (33%). Diuretics and ACE inhibitors use dropped between 2010 and 2023 by 12% and 7% points, respectively, while ARB use increased by 8% points in this period. This is also reflected in the current prescription data report of antihypertensives in Germany: even though ACE inhibitors (like Ramipril) remain the most prescribed individual agent, they are slightly declining in favor of ARBs (like Sartans) [13]. In this report, the declining prescription of diuretics and a slow increase in the prescription volume of CCBs in Germany is noted as well [13]. Internationally, there is also a clear trend toward prescribing ACE inhibitors, ARBs, and CCBs as first‐line agents [14, 15]. The declining use of diuretics has been shown in other studies as well [16, 17]. Similar shifts have been specifically reported in other countries: In the UK for instance, prescribing patterns shifted significantly following a 2007 guideline change, with ACE inhibitors and CCBs replacing diuretics and beta‐blockers as the most common initial therapies for hypertension [17]. In the US for instance, ACE inhibitors and ARB monotherapy increased between 2005 and 2016, while diuretic and beta‐blocker monotherapy decreased [14].

Evidence showed that polytherapy, particularly utilizing single‐pill combinations, is significantly more effective for achieving and maintaining BP control compared to monotherapy [18]. Moreover, due to better adherence, research revealed that single‐pill combinations of antihypertensives are more effective at achieving BP targets compared to free‐equivalent combinations [19]. In line with this evidence, ESC Guidelines recommend treatment with a single‐pill combination of two major drug classes is recommended [4]. Despite this recommendation, antihypertensive monotherapy slightly increased in Germany from 25.7% in 2008–2010 to 32.6% in 2021–2023. Two surveys of primary care practices in Germany 2016–2020 and from 2022 showed a similar order of magnitude of one third of treated hypertensive patients on monotherapy [20, 21], a similar survey from 2022 showed that 25% of patients with known hypertension were on monotherapy [22]. Meanwhile, our data show that single pill combination correspondingly decreased by 7.8% points in this period. This is in line with analyses of dispensed antihypertensive medication in Germany, which showed that only 10.9% of the prescribed packs of antihypertensive drugs in 2020 were fixed‐dose combination antihypertensives and had even decreased since 2016 [23].

The German data prescription report for 2024 showed a shift in the prescription of fixed‐combinations types (also called single pill combination‐types). Although the prescription of some specific combinations are declining (i.e., ACE‐inhibitors with diuretics), others are seeing substantial growth (such as Sartans or ACE‐inhibitors with CCBs) [13]. Still according to the 2024 report, the dominant volume of antihypertensive prescription is polytherapy with several drugs, suggesting that most patients in Germany require at least two, and often three or more, active ingredients from different drug classes to manage hypertension [13].

Although not statistically significant, our analysis showed that BP control in adults taking monotherapy (60.6%) was lower compared to BP control in adults taking polytherapy: single pill combination (67.2%) or combination therapy with several pills (64.9%). In the US for instance, the proportion of adults on monotherapy did not change between 2005 and 2016, and a high number of patients with uncontrolled BP were under monotherapy [14]. However, recent data among newly diagnosed US veterans (2000–2019) showed a significant increase in the trend of initial monotherapy [24].

In both time periods, about two out of three treated older adults with hypertension had their BP controlled. This proportion is higher than the 41% control rate reported from a large medical practice questionnaire study from 2021 [20] and 53% control from 2022 [22], but may be explained by less strict adherence to resting times, to the use of a separate room, as well as to conducting several measurements in the medical practice setting, all factors which may lead to higher measured BP and thus lower proportions of control [20]. However, our results cannot be directly compared to these studies for several reasons including different age groups and recruitment from population registries versus medical practice patients. In contrast to our previous trend analysis (1998 and 2008–2011) of similar control rates among treated men and women aged 55–79 years with hypertension [25], we found in our current analysis that women had lower BP control rates than in the previous survey and lower rates than men. Other international studies reported already before age‐dependent sex differences in BP control lower control among older women and younger men [26, 27]. Unlike our results, in the recent WHO report on hypertension, women including younger women and not only older women as in our analysis consistently demonstrate a higher hypertension control rate than men in almost every country reported [28].

We found that the overall mean SBP remained stable over time at around 132 mmHg. Recent data from Switzerland showed a mean SBP of 134.5 mmHg in individuals 65 years and older [29]. In line with our results, the study of Switzerland showed that older women with hypertension have worsening control between the time periods of 2005–2014 and 2015–2023 [29].

Even though the guidelines for hypertension treatment do not differentiate between sex [30], there might be an under‐ or inadequate treatment of older women with hypertension.

### 4.1 Strengths and Limitations

A major strength of this study is the use of nationwide samples of older adults, which enables robust population‐level comparison over give time periods. Contrary to most epidemiological studies, detailed information on medication use was obtained from ATC‐coded packages rather than relying on participant's recall. This approach significantly reduces reporting errors and provides a more accurate assessment of antihypertensive medication use and treatment patterns. Both studies used comparable and highly standardized procedure to measure BP, producing data with high levels of completeness and validity. Furthermore, the use of sampling weights ensures that the samples reflect the socio‐demographic distribution of the general population while accounting for temporal changes. As an example, participants of Gesundheit 65+ had obtained higher levels of education compared to the DEGS1 study participants 15 years ago due to the educational expansion gaining momentum following the end of World War II in Germany.

Several limitations should be mentioned. Firstly, healthier and more health‐focused individuals may be overrepresented in our samples, potentially leading to an overestimation of treatment and control rates. Secondly, treatment adherence itself cannot be directly assessed in our data. In line with standard epidemiological practice, participants underwent examination on a single occasion. Repeated or 24‐h measurements would provide more detailed information on BP. Different oscillometric devices were used, though a calibration formula addressed device‐specific differences, some residual measurement heterogeneity may remain. Nevertheless, standardization in this regard would be preferable. Further, examination locations differed (DEGS1: study centers; Gesundheit 65+: home visits), which may have influenced BP measurement conditions. Home visits in Gesundheit 65+ may have facilitated more complete reporting of medication, as participants had easier access to their prescriptions during the examination. Lastly, our population‐based samples were not large enough to assess sex specific differences in treatment approaches with adequate statistical power.

## 5 Conclusion

Antihypertensive medication is highly prevalent in the older population in Germany and is therefore an important topic both for primary care providers as well as for the older population itself. However, among treated hypertensives, the last decade did not bring any further improvement: monotherapy persists at about one third, single pill combinations are rare and even decreased compared to one decade ago and control even decreased in treated women with hypertension.

## Author Contributions

J.B., G.S., J.F., and H.N. designed the study. J.B., G.S., and H.N. conceptualized the analysis. J.B. performed the analysis. All authors had substantial input to the interpretation of the data. G.S. and J.B. drafted the manuscript and all authors critically revised it, gave final approval for the version to be submitted and agreed to be accountable for all aspects of the work.

## Funding

The DEGS1 and Gesundheit 65+ (Grant No: ZMVI1‐2518FSB410) studies were funded by the German Federal Ministry of Health and the Robert Koch Institute. These analyses were supported by the German Centre for Cardiovascular Research (DZHK).

## Conflicts of Interest

The authors declare no conflicts of interest.

## Supporting information




**Supporting File 1**: jch70250‐sup‐0001‐tableS1.docx.

**Supporting File 2**: jch70250‐sup‐0002‐tableS2.docx.

**Supporting File 3**: jch70250‐sup‐0003‐tableS3.docx.
