# Supplementary material for: Trends in Antihypertensive Medication Use and Blood Pressure Control in Adults Aged 66–79: Results of the National Examination Surveys DEGS1 and Study on Health of Older People Gesundheit 65+
Source: J Clin Hypertens (Greenwich). 2026 Apr 11;28(4):e70250. doi: 10.1111/jch.70250 (PMC13069874; doi:10.1111/jch.70250)
Supplement: Supplementary file 1 — Supporting File 1: jch70250‐sup‐0001‐tableS1.docx. [file JCH-28-e70250-s002.docx]

| Classes of medication with anti-hypertensive main effect | Anatomical Therapeutic Chemical (ATC) codes |
| --- | --- |
| Diuretics | **Monotherapy:** C03A, C03AX, C03B, C03C, C03D, C03XA, C03XP01-03 |
|  | **Polytherapy:**  C02L, C03E, C03XH, C03XP30, C07B, C07C, C07D, C08G,  C09BA, C09DA, C09DX01, C09DX03, C09XA52,C09XA54 |
| Beta-blockers | **Monotherapy:** C07A |
|  | **Polytherapy:** C07B, C07C, C07D, C07E, C07F, C07GA |
| Calcium channel blockers | **Monotherapy:** C08C, C08D, C08E |
|  | **Polytherapy:** C07FB, C08DA51, C08G, C09BB, C09DB, C09XA53,  C09XA54, C09DX01, C09DX03 |
| ACE inhibitors | **Monotherapy:** C09A |
|  | **Polytherapy:** C09B |
| Angiotensin receptor blockers | **Monotherapy:** C09CA, C09XA01, C09XA02 |
|  | **Polytherapy:** C09D, C09XA52, C09XA53, C09XA54 |
| Other antihypertensive drugs | **Monotherapy:** C02AA, C02AB, C02AC, C02AP01, C02BA, C02BB, C02C, C02D, C02KA, C02KB, C02KC, C02KD, C02KH01, C02KP01-02, C02KX |
|  | **Polytherapy:** C02AP51, C02KH20, C02KP30, C02KP52, C02L, C02N |

Supplemental Table 1. Six classes of medication with anti-hypertensive main effect according to Anatomical Therapeutic Chemical (ATC) codes
