# Supplementary material for: Trends in Antihypertensive Medication Use and Blood Pressure Control in Adults Aged 66–79: Results of the National Examination Surveys DEGS1 and Study on Health of Older People Gesundheit 65+
Source: J Clin Hypertens (Greenwich). 2026 Apr 11;28(4):e70250. doi: 10.1111/jch.70250 (PMC13069874; doi:10.1111/jch.70250)
Supplement: Supplementary file 2 — Supporting File 2: jch70250‐sup‐0002‐tableS2.docx. [file JCH-28-e70250-s003.docx]

| Sex | Diuretics | | Beta blocker | | CCB | | ACE | | ARB | | C02 | |
| --- | --- | --- | --- | --- | --- | --- | --- | --- | --- | --- | --- | --- |
|  | **2010** | **2022** | **2010** | **2022** | **2010** | **2022** | **2010** | **2022** | **2010** | **2022** | **2010** | **2022** |
| **Women** | 35.6 | 24.4 | 37.1 | 32.9 | 17.5 | 17.3 | 28.7 | 23.5 | 21.6 | 25.1 | 4.5 | 1.5 |
| 95% CI | 31.5-40.0 | 20.0 - 29.5 | 32.8- 41.5 | 26.2 - 40.4 | 14.4 - 21.1 | 12.3- 23.7 | 24.8- 32.9 | 18.0- 30.0 | 18.2- 25.3 | 18.9- 32.5 | 2.7 - 7.1 | 0.6 - 3.8 |
|  |  |  |  |  |  |  |  |  |  |  |  |  |
| **Men** | 36.9 | 25.7 | 38.48 | 32.6 | 19.45 | 22.2 | 37.0 | 27.4 | 18.2 | 30.8 | 3.6 | 3.4 |
| 95% CI | 32.9- 41.2 | 19.8- 32.8 | 33.8- 43.4 | 26.6 - 39.1 | 16.2 - 23.2 | 16.9- 28.7 | 33.1- 41.2 | 21.9- 33.7 | 15.3- 21.6 | 25.7 - 36.5 | 2.4 - 5.4 | 1.6 - 7.1 |

Supplemental Table 2 Proportion of antihypertensive use in Germany in 2010 (DEGS 2008 to 2011, n=1,041) and 2022 (Gesundheit 65+ 2021 to 2023, n=343) studies, with 95% confidence intervals (CI) by sex. Weighted to the population 2020. CCB: calcium channel blockers; ACE: angiotensin-converting enzyme inhibitors; ARB: angiotensin receptor blockers, and C02: Antihypertensive with ATC Code C02.
