# Supplementary material for: Trends in Antihypertensive Medication Use and Blood Pressure Control in Adults Aged 66–79: Results of the National Examination Surveys DEGS1 and Study on Health of Older People Gesundheit 65+
Source: J Clin Hypertens (Greenwich). 2026 Apr 11;28(4):e70250. doi: 10.1111/jch.70250 (PMC13069874; doi:10.1111/jch.70250)
Supplement: Supplementary file 3 — Supporting File 3: jch70250‐sup‐0003‐tableS3.docx. [file JCH-28-e70250-s001.docx]

|  | | **Total** | | **Monotherapy** | | **Polytherapy** | |
| --- | --- | --- | --- | --- | --- | --- | --- |
| **Sex** | **Year** | % | | % | 95% CI | % | 95% CI |
| Men | 2010 | 100% | | 22.15 | 18.10- 26.81 | 77.85 | 73.19- 81.90 |
|  | 2023 | 100% | | 31.79 | 23.65- 41.22 | 68.21 | 58.78- 76.35 |
| Women | 2010 | 100% | | 28.43 | 23.59- 33.82 | 71.57 | 66.18- 76.41 |
|  | 2023 | 100% | | 33.27 | 24.74- 43.06 | 66.73 | 56.94- 75.26 |
| **Mean SBP (in mmHg)** | | | | | | | |
|  |  |  | 95% CI |  | 95% CI |  | 95% CI |
| Men | 2010 | **134.98** | 133.31-136.66 | **137.85** | 134.59-141.10 | **134.17** | 132.37-135.97 |
|  | 2023 | **128.71** | 125.95-131.47 | **128.42** | 123.56-133.29 | **128.85** | 125.52-132.17 |
| Women | 2010 | 130.83 | 129.36- 132.29 | 133.05 | 130.55-135.55 | 129.95 | 128.04-131.87 |
|  | 2023 | 133.69 | 129.66-137.71 | 137.69 | 130.01-145.37 | 131.72 | 127.49-135.95 |
| **Mean DBP (in mmHg)** | | | | | | | |
| Men | 2010 | 82.29 | 81.11- 83.47 | 84.48 | 82.16- 86.79 | 81.66 | 80.40- 82.93 |
|  | 2023 | 82.78 | 81.18- 84.38 | 84.28 | 81.21- 87.36 | 82.06 | 80.28- 83.83 |
| Women | 2010 | 81.40 | 80.44- 82.35 | 82.83 | 81.19- 84.47 | 80.83 | 79.61- 82.05 |
|  | 2023 | 80.97 | 78.66- 83.29 | 84.18 | 80.22- 88.14 | 79.39 | 76.75- 82.04 |
| **BP control (in %)** | | | | | | | |
| Men | 2010 | 62.95 | 57.00- 68.53 | 53.37 | 40.77- 65.56 | 65.67 | 58.87- 71.89 |
|  | 2023 | 69.76 | 61.12- 77.20 | 68.10 | 48.87- 82.65 | 70.54 | 61.59- 78.14 |
| Women | 2010 | **73.76** | 68.81- 78.18 | 68.29 | 57.13- 77.67 | 75.94 | 69.67- 81.26 |
|  | 2023 | **58.00** | 47.44- 67.88 | 53.95 | 36.23- 70.73 | 60.02 | 47.07- 71.71 |

Supplemental Table 3. Mean BP and proportion of BP control among participants aged 66 to 79 years with treated hypertension in Germany in 2010 (DEGS 2008 to 2011, n=1,041) and 2023 (Gesundheit 65+ 2021 to 2023, n=343) studies, with 95% confidence intervals (CI) by sex. Weighted to the population 2020. Values printed bold if differences between studies statistically significant (p<0.05). BP control: <140/90 mmHg
